# Identification of a Tumor Specific, Active-Site Mutation in Casein Kinase 1α by Chemical Proteomics

**DOI:** 10.1371/journal.pone.0152934

**Published:** 2016-03-31

**Authors:** Eric S. Okerberg, Anna Hainley, Heidi Brown, Arwin Aban, Senait Alemayehu, Ann Shih, Jane Wu, Matthew P. Patricelli, John W. Kozarich, Tyzoon Nomanbhoy, Jonathan S. Rosenblum

**Affiliations:** 1 ActivX Biosciences, Inc., La Jolla, CA, United States of America; 2 Wellspring Biosciences LLC, La Jolla, CA, United States of America; Oak Ridge National Laboratory, UNITED STATES

## Abstract

We describe the identification of a novel, tumor-specific missense mutation in the active site of casein kinase 1α (CSNK1A1) using activity-based proteomics. Matched normal and tumor colon samples were analyzed using an ATP acyl phosphate probe in a kinase-targeted LC-MS^2^ platform. An anomaly in the active-site peptide from CSNK1A1 was observed in a tumor sample that was consistent with an altered catalytic aspartic acid. Expression and analysis of the suspected mutant verified the presence of asparagine in the probe-labeled, active-site peptide for CSNK1A1. Genomic sequencing of the colon tumor samples confirmed the presence of a missense mutation in the catalytic aspartic acid of CSNK1A1 (GAC→AAC). To our knowledge, the D163N mutation in CSNK1A1 is a newly defined mutation to the conserved, catalytic aspartic acid of a protein kinase and the first missense mutation identified using activity-based proteomics. The tumorigenic potential of this mutation remains to be determined.

## Introduction

The human protein kinases comprise a family of over 500 enzymes with key roles in diverse aspects of biology. Protein kinases govern nearly every key step involved in the modulation of cellular physiology as a result of external or internal stimuli. Numerous kinases have been implicated as causative in a variety of pathophysiologic conditions. As a result, a massive research effort has been undertaken to characterize basic kinase enzymology along with cellular and structural biology. In addition, recent efforts have determined genetic alterations in kinase genes throughout the entire genome. While several genetic alterations, including translocations and mutations, result in aberrantly active kinases, fewer inactivating genetic mutations have been observed.[1–3] Comparatively little effort has been spent characterizing proteome-wide changes in kinase expression or activity in patient-derived samples.[4–5]

**Fig 1:**
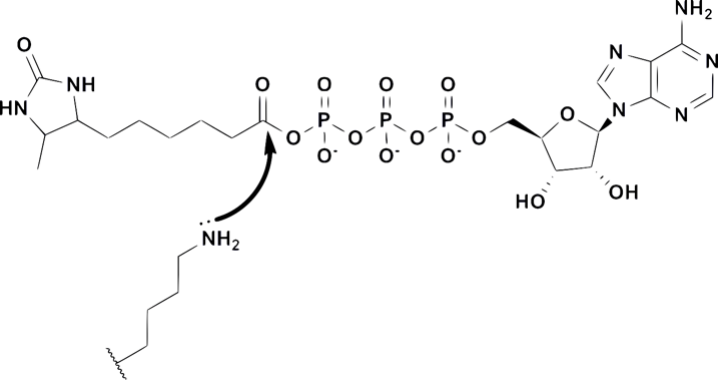
The structure of an ATP acyl phosphate probe containing a desthiobiotin affinity tag and a schematic mechanism for covalent labeling of conserved lysines in the kinase active site.

Chemical proteomics (or activity-based proteomics) describes the use of chemical probes to measure the abundance and/or enzymatic activity of specific proteins within a complex biological sample.[6–8] The probes used in such studies generally contain a covalent inhibitor or reactive group coupled to a secondary tag for selective enrichment and/or measurement of the proteins of interest (affinity tag, fluorescent tag, click tag, etc.). Acyl phosphates of ATP (Fig 1) have been developed as probes for nucleotide binding proteins including protein kinases, lipid kinases, dehydrogenases and ATPases.[9–10] These probes have proven particularly useful for measuring kinase inhibitor selectivity within complex biological samples, including target organs from inhibitor-treated animals.[11–12]

Proteins with ATP binding sites can bind probes such as the one shown in Fig 1. Conserved lysines in the binding site attack the acyl phosphate moiety, displacing ATP and leaving desthiobiotin covalently attached to the protein. ATP acyl phosphates provide such broad coverage for nucleotide binding proteins that a targeted liquid chromatography-mass spectrometry (LC-MS^2^) method was developed to focus on specific subsets of probe-labeled proteins within a given sample.[8] Samples are treated with the probe followed by denaturation, reduction, alkylation, gel-filtration, and proteolytic digestion with trypsin. Desthiobiotinylated peptides are affinity-enriched using streptavidin beads, washed to remove non-labeled peptides, and eluted for subsequent analysis.[8] The samples are analyzed using a “targeted” LC-MS^2^ approach in which the elution time and mass-to-charge ratios (m/z) of labeled peptides have been previously measured and subsequently assembled into panels of up to ~300 probe-labeled, active-site peptides per LC-MS^2^ run. Panels can be tailored for the analysis of a subset of labeled proteins (e.g. protein kinases or non-kinase ATPases) based on the relative expression levels in a specific proteome. Quantitation of peptide signal is achieved by integrating signature fragment ion intensities (MS^2^ fragment ions) derived from the previously determined m/z and elution time for a given target. This approach provides dramatic improvements in signal-to-noise (compared to parent ion extraction) and permits quantitative analysis of desthiobiotinylated lysine-containing peptides. In this report, we describe the observation of a tumor-specific, mutated CSNK1A1 active-site peptide from close examination of LC-MS^2^ data and the subsequent verification that the CSNK1A1 gene was mutated.

## Materials and Methods

### Tissue samples

Colon adenocarcinoma and adjacent normal tissue biopsies were obtained from 12 patients (Genomics Collaborative, now BioServe, Beltsville, MD). All samples were obtained anonymously and assigned random patient identifiers. The tumors varied in grade and patients had various treatments and outcomes. The same samples were previously analyzed by activity-based profiling of the serine hydrolases.[13] Sample 815zp was Stage 3 (c2) disease of the upper rectum and the tumor block subjected to profiling was 90% tumor based on histology of adjacent tissue.

### KiNativ profiling

Chemoproteomic analysis using acyl phosphates of ATP has been described extensively elsewhere.[7–10,14] The desthiobiotin ATP acylphosphate probe used in these studies is illustrated in Fig 1 and is available for purchase from Thermo scientific (cat. #88311). Colon tissue lysates were prepared in 20 mM Hepes, pH 7.5, 150 mM NaCl, 0.1% triton, with phosphatase inhibitors (A.G. Scientific) and disintegrated with ceramic beads in an Omni Bead Rupture 24 (setting 2). The resulting lysate was clarified by centrifugation for 30 min. at 16,000 g and gel-filtered using DG10 columns (Bio-Rad) that were pre-equilibrated with the lysis buffer. After gel-fitration, MnCl_2_ was added to a final concentration of 10 mM. Lysates were then labeled with the ATP acylphosphate probe at a concentration of ten micromolar for fifteen minutes, followed by reduction (6 M urea, 10 mM DTT at 65 degrees Celsius for 15 minutes) and alkylation (40 mM iodoacetamide at 37 Celsius for 30 minutes). A second gel-filtration into 2 M urea, 10 mM sodium bicarbonate was performed prior to tryptic digestion (Promega, 0.015 mg/mL for one hour at 37 Celsius). The resulting probe-labeled peptides were captured using high-capacity streptavidin resin (Thermo Scientific) followed by extensive washing using 150 μl/wash with three different wash buffers: (A) 10 times with 1% triton, 0.5% tergitol, 1 mM EDTA in PBS (B) 60 times with PBS (C) 15 times with HPLC grade water. Peptides were eluted from the streptavidin beads using two 35 μL washes with 50% CH_3_CN/water mixture with containing 0.1% TFA at room temperature.

Samples were analyzed on Thermo LTQ ion trap mass spectrometers coupled to Agilent 1100 series micro-HPLC systems. HPLC grade solvents were used in all cases and were comprised of (A) 5% ACN, 0.1% formic acid and (B) 100% ACN, 0.1% formic acid. Samples were loaded onto a pre-column peptide capTrap, desalted, and concentrated at 5% solvent B. Probe-labeled peptides were separated in 0.18 X 100 mm columns packed with 5 μm diameter, 300 Ǻ Magic C18 stationary phase (Michrom Bioresources) equilibrated at 15% solvent B. Peptide samples were then separated using a three stage linear gradient: 15–30% solvent B from 0–100 min.; 30–50% solvent B from 100–115 min.; 50–95% solvent B from 115–120 min. The column flow rate was set to 2 μl/min. The nanospray source (Thermo Scientific) was operated with the spray voltage at 1.6 kV, capillary temperature at 200°C, capillary voltage at 46 V, tube lens voltage at 120 V, and relative collision energy set to 35%.

Data were searched against the UniRef100 database using the Sequest algorithm and integrated peak areas were determined by extracting the signal for theoretical fragments ions from each specifically targeted parent ion (corresponding to a known probe-labeled kinase peptide). The calculated fold-change was determined for each sample set (normal adjacent tissue vs. tumor sample). The cumulative data set is presented as S1 Table: Colon Cancer Kinase Profiling.

### Plasmids and oligonucleotides

An expression plasmid encoding DDK-tagged human CSNK1A1 was obtained from Origene (catalogue number RC217936, corresponding to NCBI reference sequence NP_001020276.1, splice form 1). Mutagenesis of Asp 136 to Asn (GAC—>AAC) was performed at GenScript USA, Inc. (Piscataway, NJ). For PCR amplification of DNA containing the exon coding for the CSNK1A1 active-site peptide bounded by intron sequences, the following oligonucleotides were used:

CKIntFor: 5’TTTCCAGATGATCAGTAGAATTGAA (exon begins with ATG)

CKIntRev: 5’CCAAAGCAATTCTTGTCGAA (sequence completely derived from intron)

CKIntRev was also used for sequencing. To amplify a fragment that contained only the exon sequence, the following primers, which encode the first 24 and the reverse, complimented last 24 nucleotides of the exon, were used:

CSNK-For: 5’ATGATCAGTAGAATTGAATATGTG

CSNK-Rev: 5’CTTATTACAGTGACGCCCAATACC

### Molecular biology/immunoblotting

HEK293 cells were obtained from ATCC (Manassas, VA) and cultured according to ATCC’s recommendations. Plasmid transfection was performed using Lipofectamine(R) 2000 (Life Technologies, Grand Island, NY). Total transfection time was 42hr. Following transfection, cells were directly lysed using M-PER (Thermo Scientific, Rockford, IL). Proteins were separated by SDS-PAGE and transferred to nitrocellulose. Immunoblotting was performed using anti-casein kinase antibody from Thermo Scientific (catalogue number PA-17536) and anti-rabbit secondary antibody from Cell Signalling (catalogue number 7074; Danvers, MA). Immunoblot signals were detected by chemiluminescence using SuperSignal West Femto Substrate (Thermo Scientific).

PCR was performed directly on tissue samples using the Finnzymes Phusion(R) direct PCR kit (Thermo Scientific) following the manufacturer’s protocol for non-fixed biopsies with dilution. Unpurified, heterogeneous PCR products were either directly sequenced using the downstream primer or TA-cloned and sequenced with a plasmid-specific downstream primer (all cloning and sequencing done at GeneWiz, South Plainfield, NJ).

## Results

We set out to determine whether specific kinases are altered in patient-derived colon tumor samples by using ATP acyl phosphate probes. Matched colon samples (normal and tumor) for twelve cancer patients with varying degrees of disease progression were obtained. Replicate samples from both the normal and tumor material from each donor were prepared and analyzed using an ATP acyl phosphate probe and a kinase-focused target list. Across this data set, a total of 380 kinase labeling sites (corresponding to labeling of active site and/or activation loop lysines) from 259 distinct kinases yielded quantifiable signal. A number of tumor specific changes in kinase labeling were observed (S1 Table: Colon Cancer Kinase Profiling). For example, several kinases with consistently more signal in the tumor samples relative to the matched controls include: AurA, CHED (CDK13), JAK3, p38α, CHK1, ITPK. Across the complete data set, the average CSNK1A1 signal was four-fold higher in the tumor samples relative to control. One noteworthy observation is that while p38α active site labeling is unaltered, a labeling site indicative of the p38-MAPKAPK2/3 interaction generates 6.9-fold more signal in the tumor samples relative to control.[14] While some of the altered targets have been implicated in tumorigenesis, their specific impact on tumorigenesis within the colon will likely be the subject of continued research. In addition, the potential for one or more of these targets to serve as a useful biomarker also exists. Finally, a recent report describes the use of ATP acyl phosphates for kinase profiling of lung tumors relative to normal, adjacent control tissue and is noteworthy for comparison. [15]

**Fig 2:**
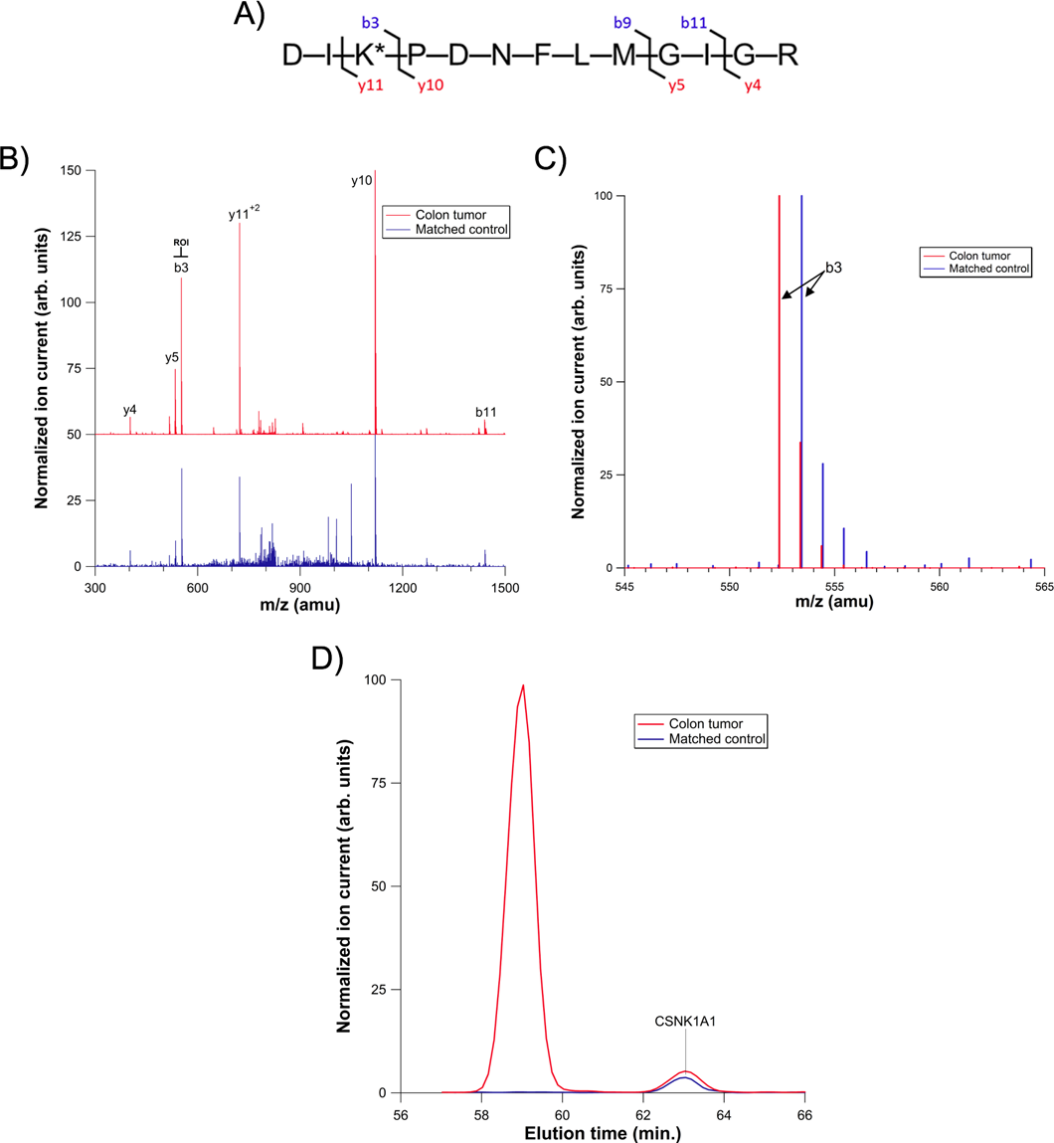
Analysis of a CSNK1A1 active-site peptide in both colon tumor and matched control samples. A) Amino acid sequence of the wild type peptide. B) LC-MS/MS spectra for the CSNK1A1 peptide. The colon tumor sample is off-set in the Y-axis by 50 units for clarity and the region of interest (ROI) is highlighted. C) Region of interest in the LC-MS/MS spectra for colon tumor and matched control samples. Each spectrum is normalized to the highest intensity ion. D) Extracted ion chromatograms for CSNK1A1 from both colon tumor and matched control samples. Signals are normalized to the peak eluting at 59.8 minutes.

An unusual circumstance was observed for CSNK1A1in the tumor sample derived from one patient, sample 815zp (Fig 2). Under our experimental conditions, the CSNK1A1 peptide elutes at 63.0 minutes, but signal apparently corresponding to CSNK1A1 was observed at both 59.8 minutes and 63.0 minutes in the tumor samples of this patient. The observed elution time shift of ~3 minutes for the first extracted ion peak is well outside our expected standard deviation for elution times of ± 0.98 minutes. Moreover, in the 59.8 minute extracted ion peak, a fragment ion in the MS^2^ spectrum corresponding to the peptide sequence “DIK*” (where K* is the desthiobiotinylated lysine) of CSNK1A1 was shifted from the expected m/z of 553.4 to 552.4 atomic mass units (amu). This fragment ion contains both the conserved catalytic aspartic acid (D) and an adjacent, probe-labeled lysine (K*). This “DIK” (or the mass equivalent “DLK”) sequence is largely conserved among protein kinases, and the 553.4 amu fragment can be diagnostic for the presence of a probe-labeled kinase peptide. All fragment ions that did not contain the catalytic aspartic acid were unchanged, which suggested that a specific alteration was present in fragments containing the aspartic acid from “DIK*”. The observed mass shift was reproduced on replicate analyses, confined to CSNK1A1, and was not present in any normal colon samples, demonstrating that the one amu shift was not a simple artifact of sample procurement, processing, etc. Moreover, the observed one amu mass shift was consistent with a tumor-specific missense mutation (D→N), and a similar elution time shift had previously been observed in our labs upon mutation of a kinase active site aspartic acid to asparagine (in a different kinase). As such, we sought verification of a missense mutation through probe-based analysis of a recombinant CSNK1A1 D→N mutant.

**Fig 3:**
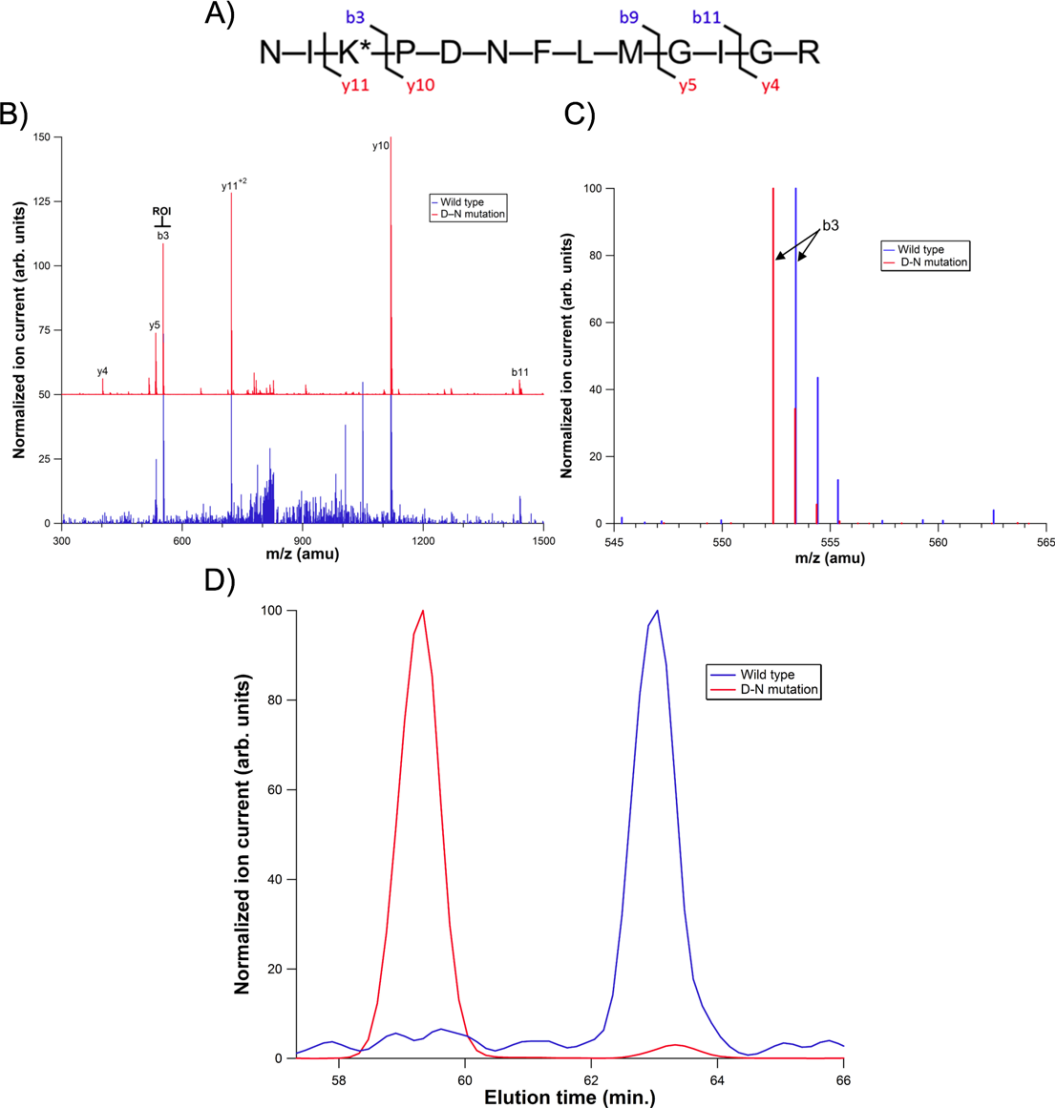
Analysis of a CSNK1A1 active site peptide from wild-type and mutant CSNK1A1 expressed in HEK293 cells. A) Amino acid sequence of the mutant peptide. B) LC-MS/MS spectra for wild-type and mutant protein. The mutant sample is off-set by 50 units for clarity and the region of interest is highlighted. C) Region of interest in the LC-MS/MS spectra for colon tumor and matched control samples. Each spectrum is normalized to the highest intensity ion. D) Extracted ion chromatograms from HEK293 lysates transfected with either wild-type or mutant CSNK1A1. The wild-type sample is normalized to the peak eluting at 63.0 minutes and the mutant sample is normalized to the peak eluting at 59.8 minutes.

Wild-type CSNK1A1 and CSNK1A1 D136N were transiently expressed in HEK293 cells and analyzed as the colon samples were. The observed MS^2^ spectrum and elution time for the engineered CSNK1A1 mutant were identical to that observed in the colon tumor sample (Fig 3). This verified that, in the tumor sample, the catalytic amino acid for CSNK1A1 had been converted to an asparagine from an aspartic acid.

**Fig 4:**
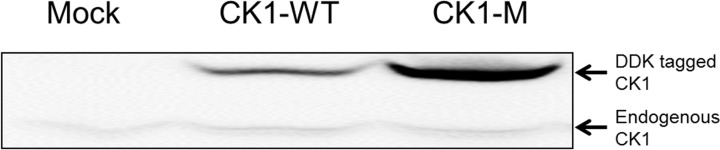
Immunoblot of recombinant CSNK1A1 samples. HEK293 cells were transfected with either no DNA (Mock), **CSNK1A1** wild type or D136N plasmids (CK1-WT and CK1-M, respectively).

The engineered enzyme also enabled an estimation of the ratio of mutant to wild type CSNK1A1 in the tumor sample. In the recombinantly expressed, engineered CSNK1A1 mutant sample, the ratio of engineered mutant to endogenous, wild-type enzyme was 8:1, as determined by western blot (Fig 4, third lane). The ratio for extracted-ion signal intensity was 80:1 (mutant:wild-type) by LC-MS^2^ analysis (Fig 3D, red trace), indicating that the mutant enzyme results in approximately 10-fold more LC-MS^2^ signal on a per mole basis, presumably due to improved ionization efficiency for asparagine relative to aspartic acid.[16] Therefore, based on the ratio of mutant:wild-type LC-MS^2^ signals (Fig 2D, red trace), the colon tumor sample contains approximately 3-fold more mutant CSNK1A1 than wild-type.

To distinguish whether the asparagine in the CSNK1A1 active site peptide was genetically encoded or the result of a post-translational event, we sequenced genomic DNA from the tumor sample presenting the apparent mutation. Two methods were used to sequence genomic DNA: direct sequencing of heterogeneous, intron-bounded sequence and cloning an exon-limited DNA fragment, followed by sequencing of individual clones. These approaches gave complementary results and clearly established that patient 815zp had a genetically encoded CSNK1A1 D136N mutation.

**Fig 5:**
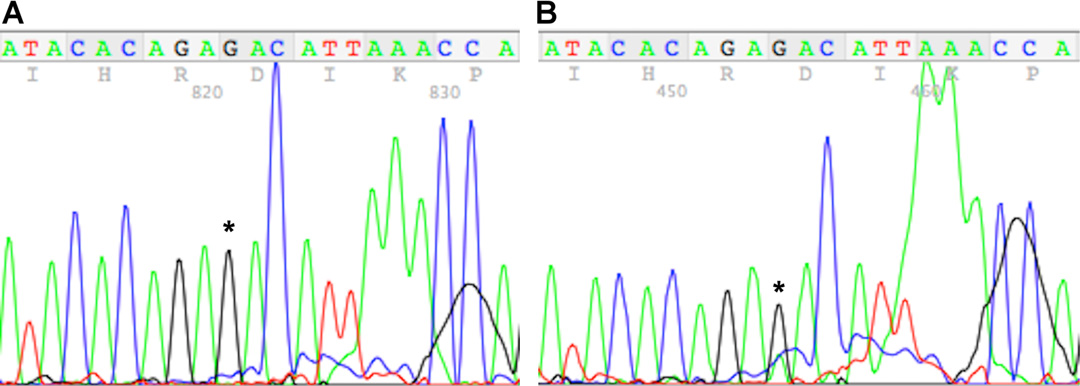
Genomic DNA sequencing of CSNK1A1. A) PCR sequencing results for control using intronic primer. B) PCR sequencing results for patient 815zp using an intronic primer. In both cases, nucleotide of interest is highlighted with an asterisk.

To sequence the heterogeneous genomic DNA, two primers were designed that anneal to intronic sequences on either side of the exon that contains the CSNK1A1 active-site peptide. PCR product was generated from a tumor that did not give signal for the mutant in the mass spectrometry experiment as well as patient 815zp. Direct sequencing of PCR product from the control tumor yielded the expected GAC codon for the active site aspartic acid. In contrast, sequencing the PCR product from patient 815zp yielded a small, but distinct peak of A in the first position of this codon concomitant with a smaller G peak relative to the control tumor (Fig 5, compare the height of the highlighted guanosine to the adjacent adenosines in panels A and B). Replacing G with A at this position would result in an AAC/asparagine codon. Though highly suggestive of a mutation, sequencing of the PCR product using this approach yielded chromatograms that were too noisy to prove the existence of the mutation.

**Fig 6:**
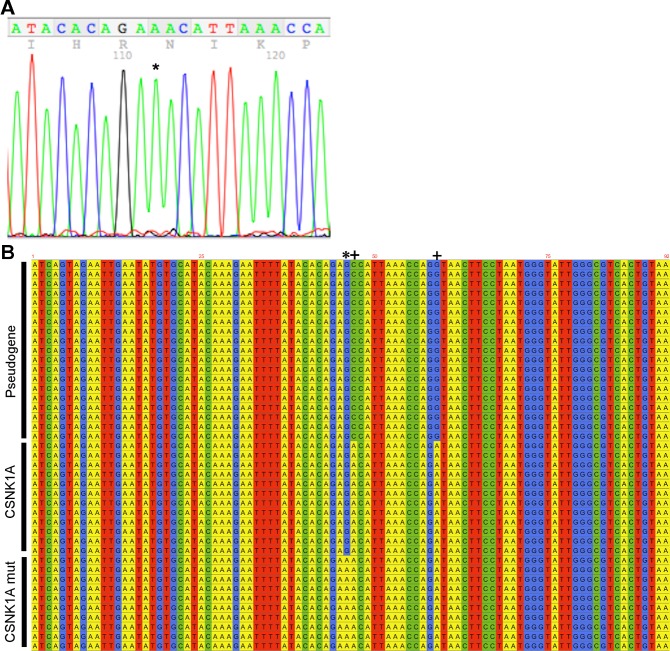
Sequence analysis of cloned PCR products from the 815zp tumor sample. A) A representative clone showing the mutated nucleotide (*). B) Alignment of all 41 sequences. Differences between pseudogene and CSNK1A1 are indicated by a plus symbol.

In order to prove the existence of a mutation at this position, primers annealing to the ends of the active-site containing exon were used to amplify and clone the exon. Several dozen clones were individually sequenced, resulting in the unambiguous identification of an AAC codon in CSNK1A1 in the 815zp tumor sample (Fig 6A). The active-site exon of CSNK1A1 is almost exactly duplicated on chromosome 15 in a CSNK1A1P1 pseudogene. As a result, this approach resulted in the sequencing of a number of pseudogene sequences (Fig 6B). Interestingly, the pseudogene is also modified at the codon for amino acid 136, in this case the GCC codes for a catalytically incompetent alanine (Fig 6B, plus symbol adjacent to the asterisk). Of the 41 clones sequenced, 19 were derived from the pseudogene, almost exactly the 1:1 ratio to CSNK1A1 expected based on their equal chromosomal representation. Of the 22 CSNK1A1 clones sequenced, 10 were from the mutant. We have not validated the ability of this assay to quantify the relative abundance of the different sequences, but the mutant sequence likely made up a substantial fraction of the tumor from patient 815zp.

## Conclusions

Numerous approaches have been used to characterize genomic and proteomic alterations in cancer. Taking advantage of conserved lysines in kinase ATP-binding sites, we have used a probe ATP acyl phosphate probes to quantitatively desthiobiotinylate kinases in normal and cancerous colon tissue. In the course of these studies, we detected an active-site mutated **CSNK1A1**. Although the mutated sequence was not in the database used for mass spectrometric identification, it gave rise to characteristic changes in the observed spectrum that enabled our correct identification. Sequencing of genomic DNA verified that the mutation was genetically encoded.

Genetically encoded, cancer promoting mutations in kinase enzymes are well documented. Most are enzymatically activating and lead to aberrant kinase activity through modification of gatekeeper residues, activation loops, auto-inhibitory domains, etc. In fact, some drug development efforts now specifically target mutated enzymes to address acquired drug resistance resulting from administration of therapeutic kinase inhibitors.[17–18] Inactivating mutations have been demonstrated in LKB1, DAPK3 and PKC and have helped define the tumor suppressor role of these enzymes.[19–22] LKB1 is unique in that it is, to our knowledge, the only other kinase with a clinically observed mutation to the strictly conserved catalytic aspartic acid of protein kinases. The active site mutation of CSNK1A1 defined here may similarly provide a mechanism for enhanced cancer cell proliferation and possible role for CSNK1A1 as a tumor suppressor.

The casein kinases were among the first kinases characterized, are ubiquitously expressed in eukaryotes, and have numerous functions.[23] One of the key roles of **CSNK1A1** is to control, in a regulated fashion, the abundance of the proliferation promoting protein beta-catenin.[24] Inactive casein kinase 1α could lead to under phosphorylation of beta-catenin, resulting in increased beta-catenin stability and signalling which could drive unregulated growth promotion. In fact, it has been observed that small molecule-mediated activation of CK1α decreases proliferation[25] whereas targeted deletion of CK1α, when combined with p53 or p21 deletion, enhances proliferation resulting in small bowel carcinoma.[26] It is possible that the D163N mutation characterized in patient 815zp led to enzymatic inactivation of CSNK1A1 and ultimately promoted, perhaps when combined with other mutation(s), the development of colon cancer.

## Supporting Information

S1 Table Colon Cancer Kinase Profiling. (XLS)
