# Correction: Identification of a Tumor Specific, Active-Site Mutation in Casein Kinase 1α by Chemical Proteomics

**DOI:** 10.1371/journal.pone.0172649

**Published:** 2017-02-15

**Authors:** Eric S. Okerberg, Anna Hainley, Heidi Brown, Arwin Aban, Senait Alemayehu, Ann Shih, Jane Wu, Matthew P. Patricelli, John W. Kozarich, Tyzoon Nomanbhoy, Jonathan S. Rosenblum

The mutation name D163N appears incorrectly throughout the article. The correct mutation name should be D136N.
